# Ultrasound-Assisted Anatomic Posterior Cruciate Ligament Reconstruction With Remnant Preservation by Reference to Distal Reflection of the Posterior Capsule and Posterior Septum

**DOI:** 10.1016/j.eats.2025.103559

**Published:** 2025-04-29

**Authors:** Xiaoqian Men, Shengkun Wu, Zhikuan Li, Yi Zheng, Yingzhen Niu, Jiangtao Dong

**Affiliations:** Third Hospital of Hebei Medical University, Hebei, China

## Abstract

Compared with anterior cruciate ligament reconstruction, posterior cruciate ligament (PCL) reconstruction has a higher failure rate and revision rate, which is associated with multiple factors such as graft option, fixation method, postoperative rehabilitation, and management of concomitant injuries. Among them, “killer turn” is a key factor in the failure of PCL reconstruction. Although there are various techniques for PCL reconstruction, the ideal surgery remains controversial. Combined with our clinical experience, ultrasound-assisted positioning is used to locate the tibial tunnel with the distal posterior capsule reflection and the posterior septum as reference to safely achieve anatomic remnant preservation and PCL reconstruction. This technique not only preserves the integrity of the posterior septum but also minimizes the impact of the “killer turn” on the graft by keeping the tibial tract as low as possible and effectively improves the success rate of PCL reconstruction.

The posterior cruciate ligament (PCL) is a main stabilizing structure to limit posterior tibial translation, and PCL injuries can lead to various degrees of sports disorders in patients. Although PCL reconstruction has been reported to improve knee instability, the high failure and revision rates should not be ignored. Surgery failure is closely associated with a variety of factors, such as graft option, fixation method, postoperative rehabilitation, and management of concomitant injuries, which not only affect the patients’ daily lives and exercise level but also may increase the risk of osteoarthritis.^1^

Currently, different techniques have been developed for PCL reconstruction,^2^ such as single-bundle reconstruction and double-bundle reconstruction, the transtibial technique and the tibial inlay technique, retaining the remnant PCL, and removing the remnant PCL. Although each of these techniques has its own advantages, the medical community has still not reached a consensus on which surgical technique is the most ideal.

We propose to conduct single-bundle reconstruction with PCL remnant preservation by using the posterior capsule reflection and posterior septum as the reference for the tibial tunnel with the assistance of ultrasound. This can ensure that the tibial tunnel is as low as possible, based on patient safety, which can largely reduce the damage to the graft caused by the “killer turn” and improve the success rate of PCL reconstruction.^3^ The femoral tunnel takes the femoral anterolateral bundle of the PCL as the reference marker.

## Surgical Technique

### Patient Positioning

The patient is placed in the supine position and a thigh tourniquet is applied. The range of the knee motion should be between 0° and 120°. A preoperative physical examination of the knee is carried out when the patient is under anesthesia, and it is compared with the postoperative physical examination to evaluate surgical outcome.

### Graft Harvest and Preparation

**Fig 1:**
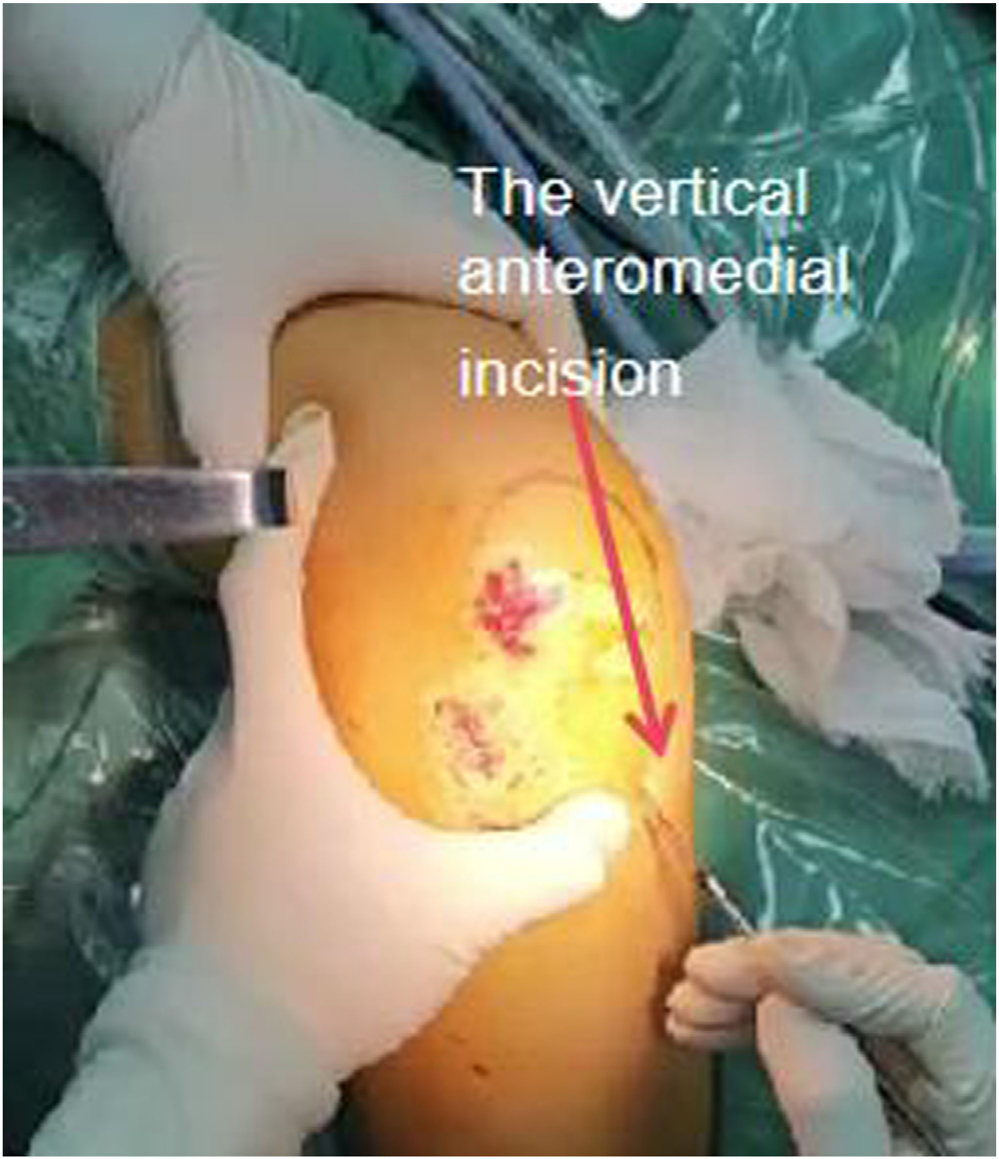
The vertical anteromedial incision at the level of the tibial tuberosity (side, right; position, flex at 90°).

The hamstring tendon (HT) is used as a graft in this technique. When the knee joint is flexed at 90°, a vertical anteromedial incision (Fig 1) at the level of the tibial tuberosity is used to expose the sartorius fascia and pes anserinus covering the HT. An incision is made on the sartorius fascia to expose the HT, and the tendon is stripped under tension (Video 1).

**Fig 2:**
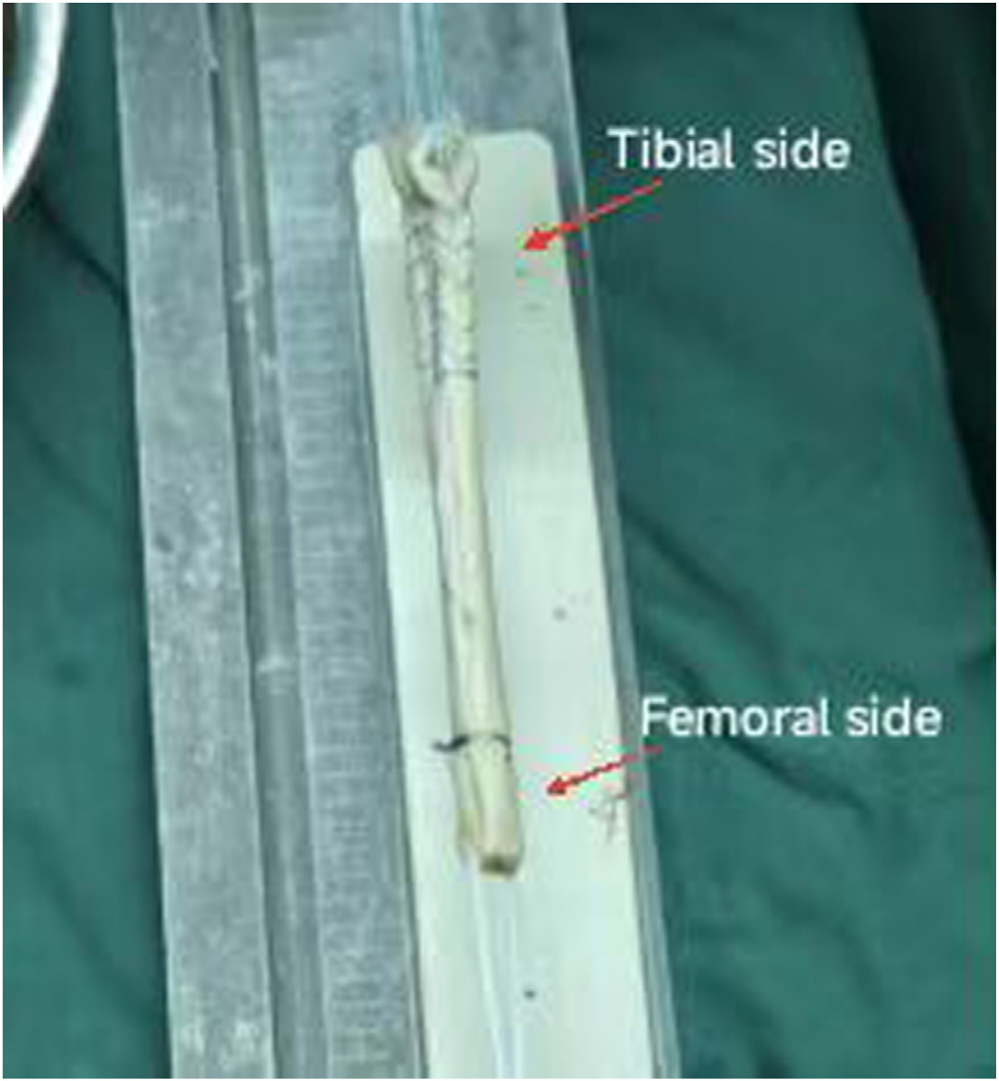
Final hamstring tendon graft for later graft passage and fixation.

Use a file to remove the excess muscle tissue and other parts on the graft, and then press the tendon into a flat shape and measure its length. Fold the tendon in half and use a tendon gauge to ensure that the diameter of the graft is between 7 and 8.5 mm. On the tibial side of the graft, use FiberWire (Arthrex) to suture both ends of the tendon for a length of 3.5 cm. Pass the adjustable looped titanium button through the femoral side of the graft with the suture thread, and fix a loop as a proximal marker 20 mm proximal to the femoral end of the graft (Fig 2). After the graft is prepared, make sure that the edges of the graft are smooth so that it can pass through the bone tunnels on both sides more easily.

### PCL Reconstruction

**Fig 3:**
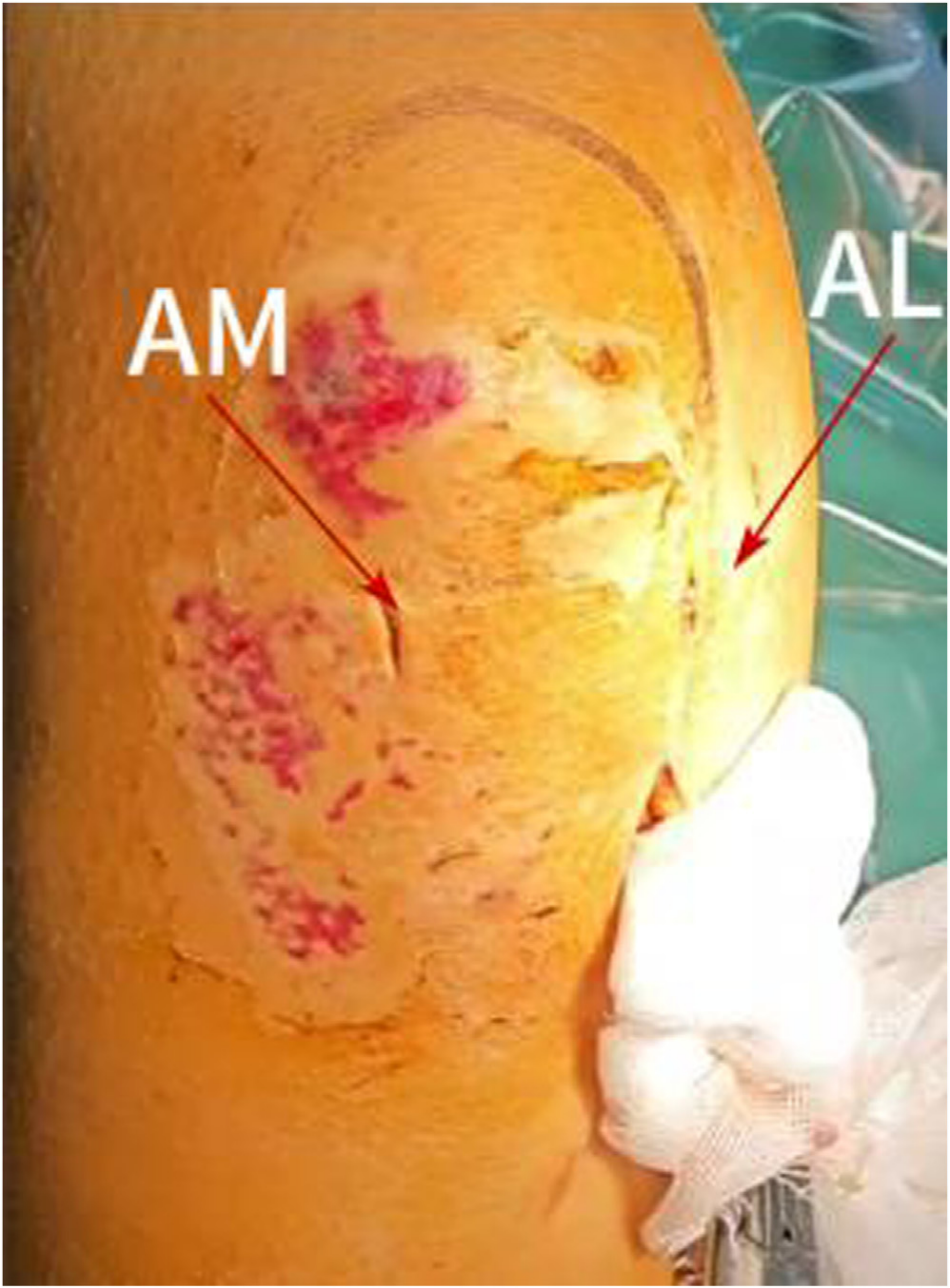
The anteromedial (AM) and anterolateral (AL) portals are established (patient side, right; position, supine).

**Fig 4:**
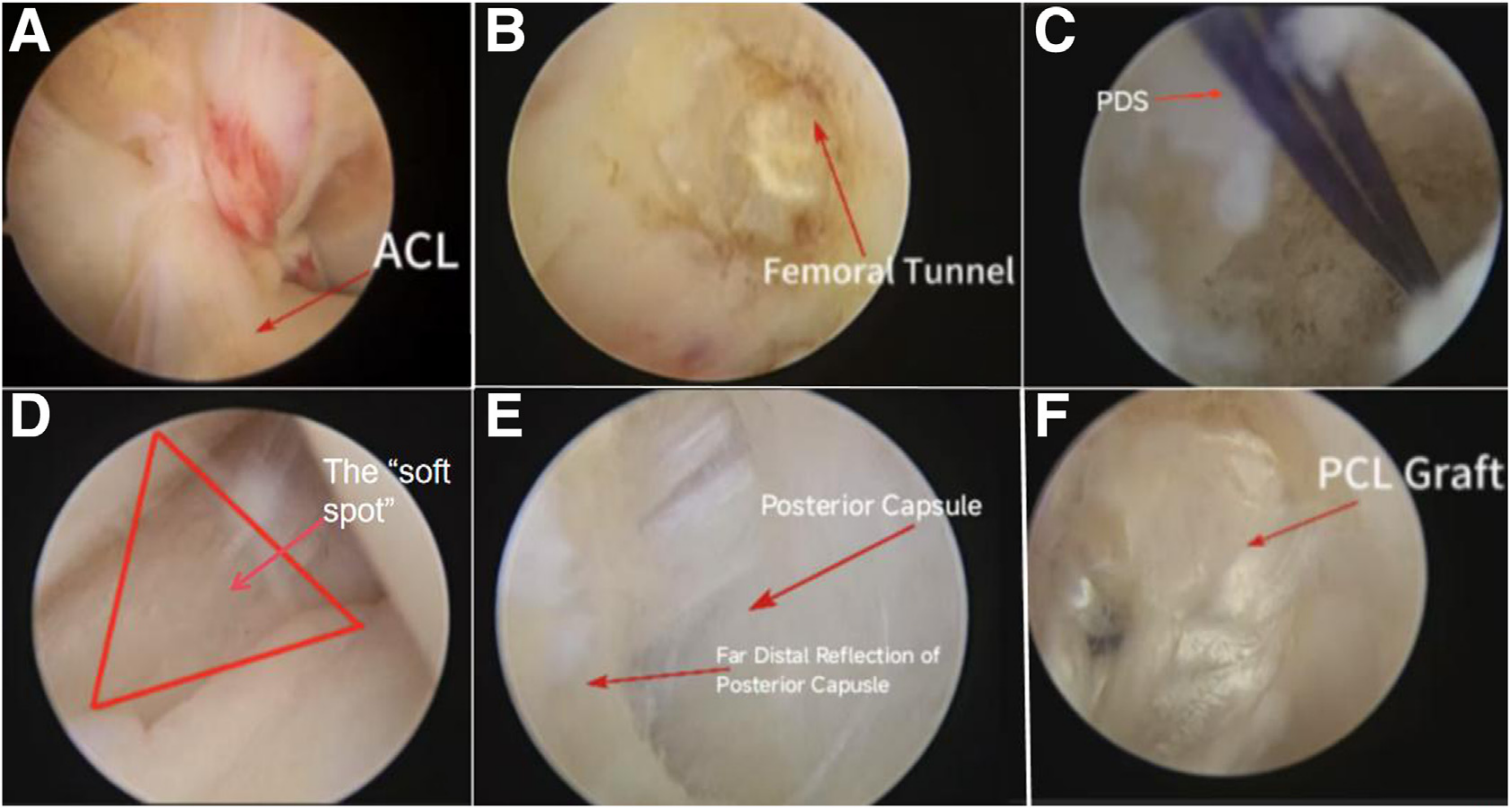
(A) Using a planner to explore the anterior cruciate ligament and posterior cruciate ligament (side, right; position, supine; anteromedial). (B) A radiofrequency probe is used to clear the tissue at the femoral insertion site of the anterolateral bundle of the posterior cruciate ligament (side, right; position “4”; anteromedial). (C) A PDS suture (Ethicon) is retained near the medial opening of the femoral tunnel (side, right; position “4”; anteromedial). (D) The triangle is enclosed by the fold of the medial head of the gastrocnemius muscle (side, right; position “4”; anteromedial). (E) The distal reflection of the posterior capsule and medial side of the posterior septum (side, right; position “4”; low posteromedial). (F) Proximal marker on the femoral side of the graft (side, right; position “4”; low posteromedial). (ACL, anterior cruciate ligament; PCL, posterior cruciate ligament.)

The anteromedial (AM) and anterolateral (AL) portals are established (Fig 3) for routine arthroscopic examination (the patient’s anterior cruciate ligament was tortuous) (Fig 4A, Video 1).

The arthroscope is inserted through the AM portal for observation, while the AL portal is used as the operative portal. When establishing the femoral tunnel, the center of the medial opening of the tunnel is 7 to 8 mm away from the distal side of the cartilage. Then, a radiofrequency probe is used to clear the tissue at the femoral insertion site of the AL bundle of the PCL (Fig 4B) to expose the medial opening of the tunnel. The femoral drill bit is placed at the center of the medial opening of the tunnel, and the femoral tunnel is drilled. The femoral tunnel consists of 2 parts: a thick tunnel at the medial opening and a thin tunnel at the lateral opening. The diameter of the thick bone tunnel is the same as that of the graft, with a length of 2 cm. After the femoral tunnel is established, a PDS suture (Ethicon) passes through femoral tunnel along the approach between the PCL and intercondylar fossa, and the PDS suture is retained near the medial opening of the femoral tunnel (Fig 4C).

**Fig 5:**
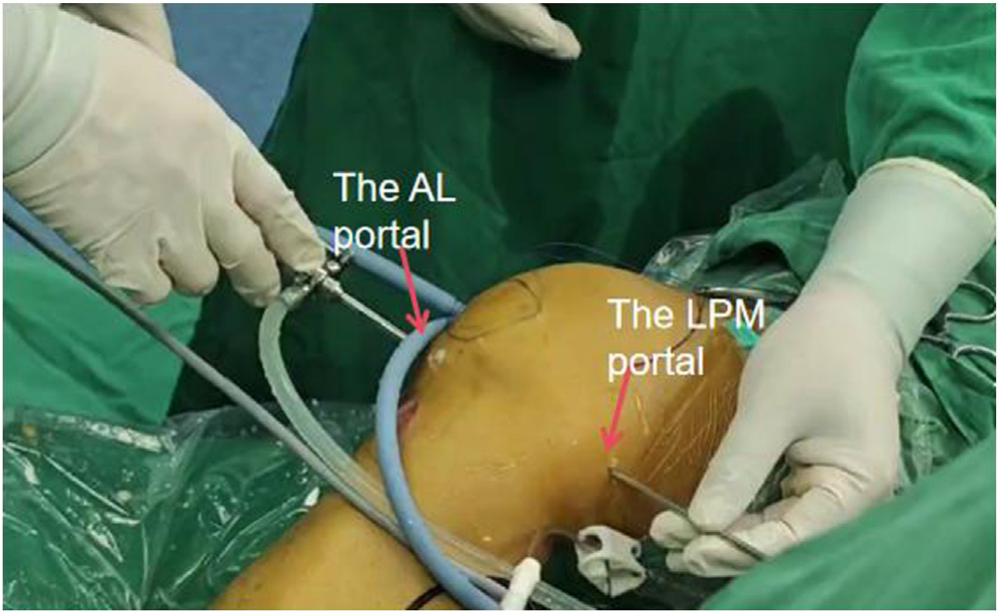
The arthroscope is removed from the anterolateral portal and transferred to the posteromedial compartment through the low posteromedial portal via a switching rod (side, right; position “4”). (AL, anterolateral; LPM, low posteromedial.)

**Fig 6:**
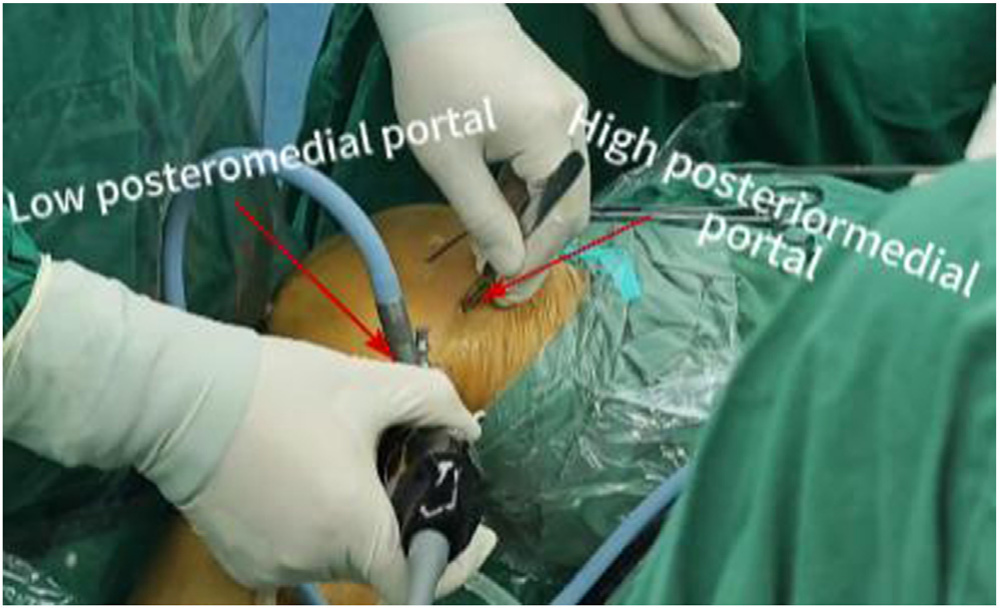
The high posteromedial portal is opened 3 to 4 cm above the low posteromedial portal (side, right; position “4”).

A tibial tunnel is established with a low posteromedial (LPM) portal and a high posteromedial (HPM) portal. The arthroscope is inserted into the posteromedial compartment through the approach between the PCL and the intercondylar fossa. In the triangular area surrounded by the ramp area of the meniscus, posterior femoral condyle, and medial head of the gastrocnemius muscle fold, the “soft spot” with the most obvious deformation is located (Fig 4D). A small skin incision is made with a sharp knife to establish the LPM portal. The arthroscope is removed from the AL portal and transferred to the posteromedial compartment through the LPM portal via a switching rod (Fig 4, Fig 5). Under direct vision, the HPM portal is opened 3 to 4 cm proximal to the LPM portal (Fig 6). The planner is inserted into the posteromedial compartment through the HPM portal to clear tissue between the posterior capsule and remnant PCL, gradually exposing the distal reflection of the posterior capsule and the medial side of posterior septum (Fig 4E).

**Fig 7:**
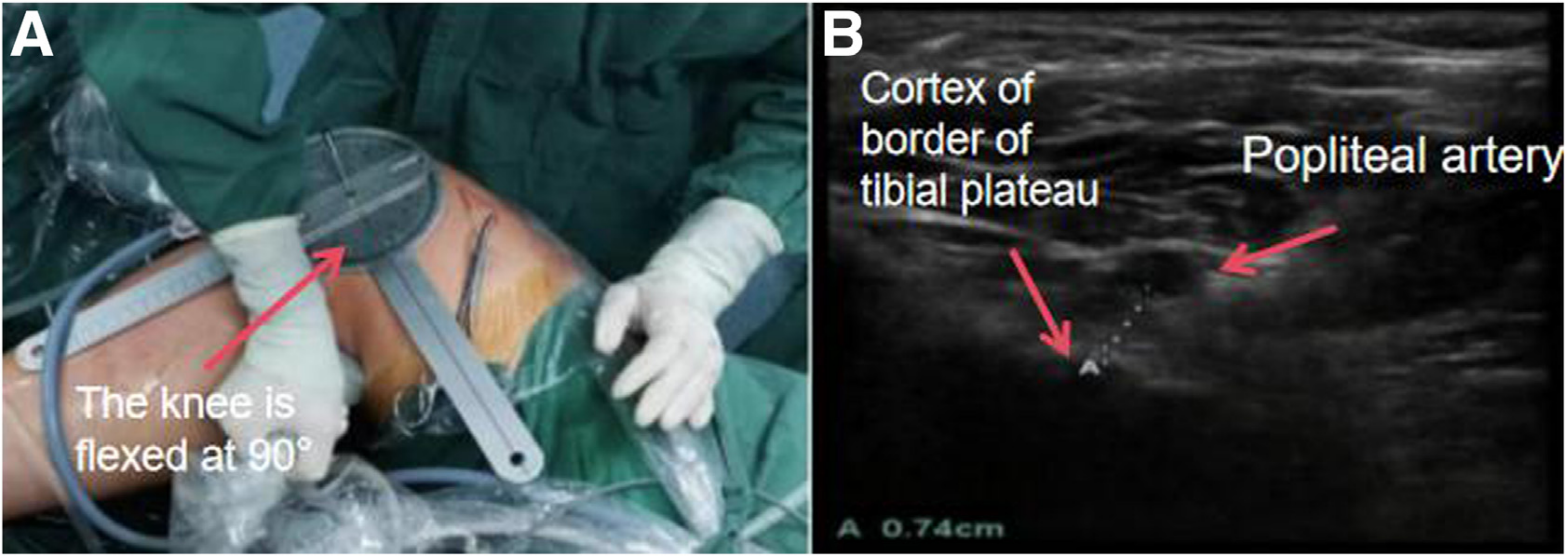
(A) The patient’s knee flexed at 90°. (B) Ultrasound probe is used to measure the distance on the horizontal axis (side, right; position “4”).

With the patient’s knee flexed at 90°, after injecting water into the joint cavity (Fig 7A), an ultrasound probe is used to measure the distance on the horizontal axis (Fig 7B). Make sure that the bone tunnel is located in the area above the champagne drop-off, and observe the distance between the position of the bone tunnel and the popliteal artery to ensure the safety of surgery.

A PCL tibial positioner is inserted through the AM portal. The central positioning point of the PCL tibial tunnel is located right above the lowest point of the distal reflection of the posterior capsule within the posteromedial compartment space and close to the medial side of the posterior septum. After the tibial tunnel is established, a radiofrequency probe and a shaver are used to clear the small amount of tissue around the bone tunnel through the tibial tunnel to ensure the patency of the bone tunnel.

### Graft Passage and Graft Fixation

**Fig 8:**
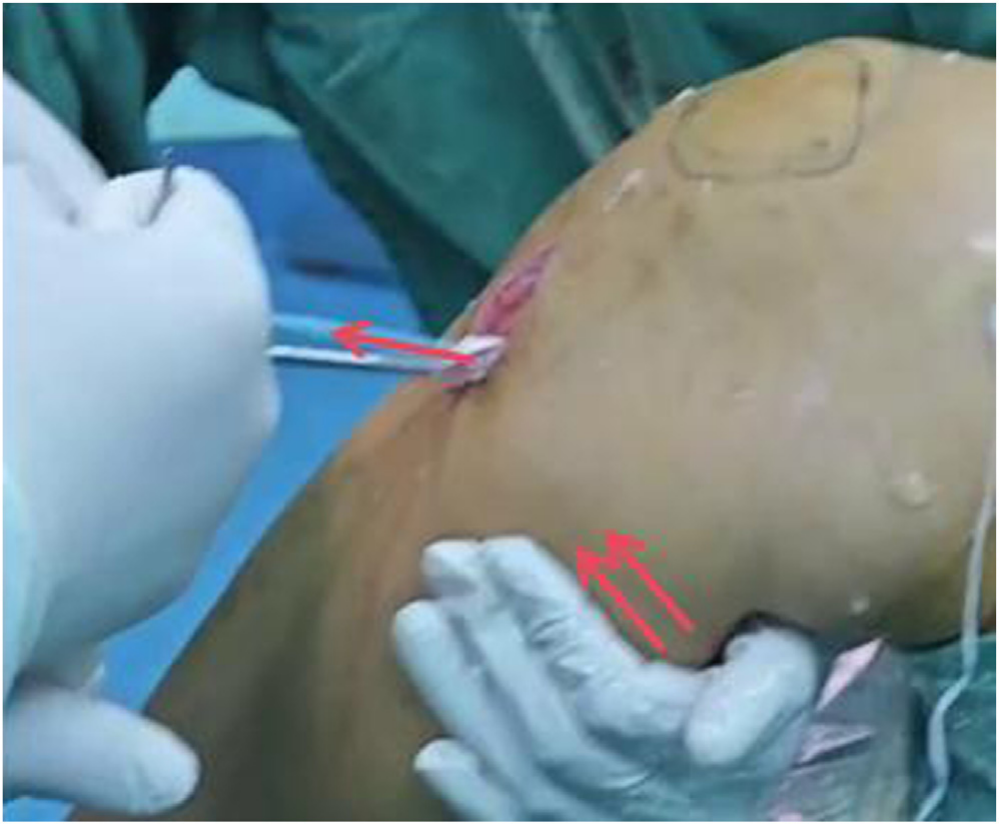
Knee in neutral position and flexed at 90°. Anterior drawer force is performed (Side, right; position, flex at 90°).

The graft is inserted through the tibial tunnel. Arthroscopy is inserted through the LPM portal, and a conical prying instrument is inserted through the HPM portal to pry open the traction suture of the graft to prevent it from getting entangled with the remnant PCL. An adjustable looped plate on the femoral side of the graft is pulled out from the lateral opening of the femoral tunnel through the PDS suture until the proximal marker on the femoral side of the graft completely enters the medial opening of the femoral tunnel (Fig 4F). With the knee in neutral position and flexed at 90°, anterior drawer force is performed to push the proximal tibia forward and tighten the graft to ensure the tension of the graft (Fig 8). The 4 sutures on the tibial side of the graft are threaded into the guidewire, and compression fixation is carried out using an interference screw. After the 4 sutures at the tibial end of the graft are fixed by the interference screw, they are threaded onto the outside row. The 4 sutures are tightened, and the outside row of pins is driven into the holes below the lateral opening of the tibial tunnel for fixation.

### Postoperative Care

After surgery, patients need to wear a knee brace to ensure the extension of their knee. On the first day after surgery, patients are encouraged to perform straight-leg raising exercises and touch-down weightbearing. In the third week after surgery, the amount of training is gradually increased. Six months after the operation, the patient is allowed to participate in sports activities.

## Discussion

Anatomic studies have found that all fibers of the tibial insertion site of the PCL are located on the medial side of the posterior septum, and the posterior septum is the lateral edge of the tibial insertion of the PCL. Therefore, by establishing the tibial tunnel with the medial edge of the posterior septum as reference in our technique, it is possible to preserve the entire remnant of the PCL to maintain the integrity of the posterior septum. Moreover, the posterior septum contains a blood supply to help restore knee function in patients after PCL reconstruction.^4^ When using the area above the distal reflection of the posterior capsule and the medial side of the posterior septum as reference for positioning the tibial tunnel, its position on the axis of the tibial plateau is similar to that reported by Lin et al.^5^ It is located slightly lateral to center of the tibial insertion of the PCL, which can effectively avoid collisions between the graft and the medial side of the intercondylar fossa and reduce failure rate of the graft.^6^

The “killer turn” is an important factor contributing to graft failure in PCL reconstruction. Previous studies have demonstrated that establishing a low tibial tunnel in PCL reconstruction can effectively reduce the effect of the “killer turn” on the graft and decrease the risk of graft tear. Biomechanical tests have shown that the low tibial tunnel has better biomechanical properties after PCL reconstruction.^7^ However, there is no unified positioning standard for the low tibial tunnel positioning technique, and the positioning methods lack reproducibility. With the assistance of ultrasound, our technique can establish the tibial tunnel as low as possible to preserve the integrity of the remnant PCL and posterior septum. Thus, it can reduce the effect of the “killer turn,” relieving the load on the graft. Meanwhile, application of ultrasound technology for positioning makes positions of the posterior capsule and posterior septum more accurate, thereby ensuring the safety of the patients and the reproducibility of the surgical technique.

**Fig 9:**
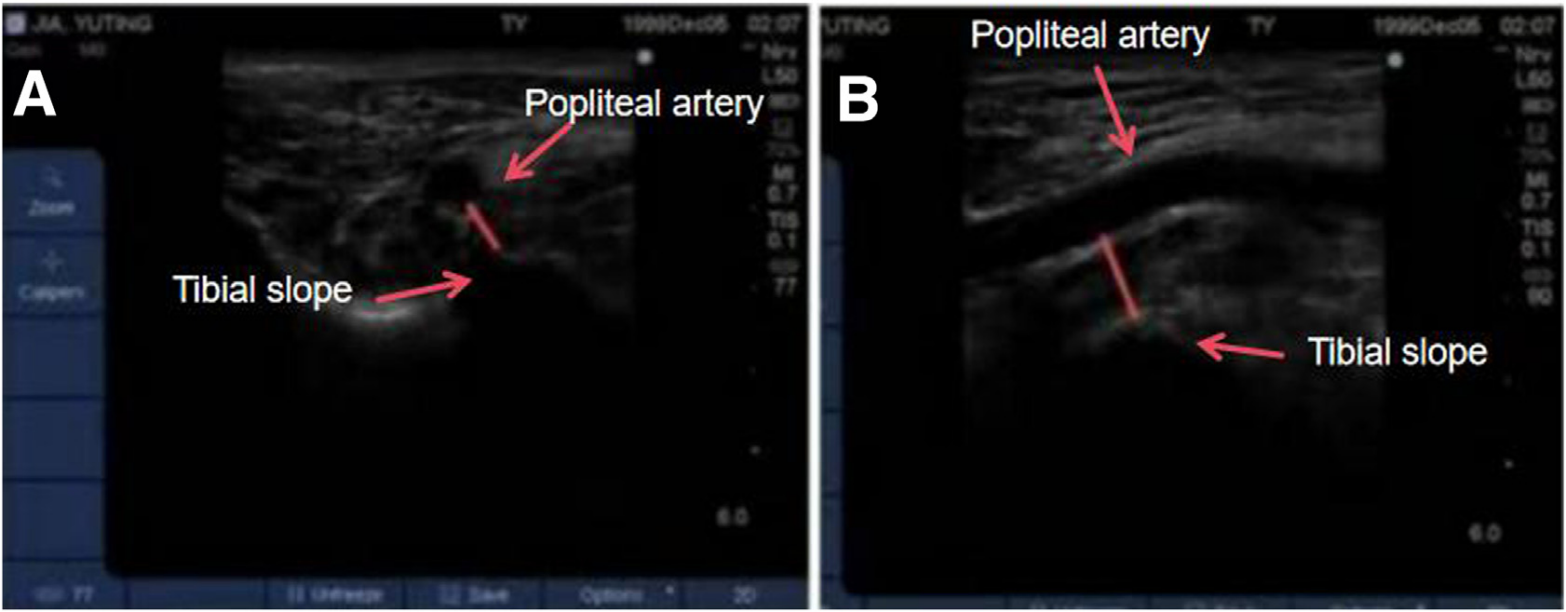
(A) Horizontal and (B) vertical modes of ultrasound positioning with the knee flexed at 90° (side, right; position “4”).

When positioning the tibial tunnel, we adopt the technique combining the HPM portal and LPM portal with the assistance of ultrasound. Our team has already conducted a preliminary experiment to verify the feasibility and safety of ultrasound-assisted positioning. We measure the distance between the posterior cortical cortex of the tibial and popliteal arteries with the assistance of an arthroscope during surgery, in both the horizontal (Fig 9A) and vertical (Fig 9B) modes. Patients who undergo PCL reconstruction using this positioning method demonstrate good surgical outcomes in the postoperative functional score and the examination with the Ligs digital joint ligament physical examination instrument.

Table 1 lists the advantages of positioning based on the distal reflection of the posterior capsule and posterior septum. Table 2 lists the benefits of preserving the remnant PCL. Table 3 lists the advantages of ultrasound-assisted positioning. Table 4 lists the limitations of this technique.

**Table 1:** Advantages of Positioning Based on the Distal Reflection of the Posterior Capsule and Posterior Septum

1. A low tibial tunnel can reduce the effect of the “killer turn” on the graft. 2. The intact posterior septum is preserved, providing a blood supply to help postoperative recovery of patients. 3. The center of the tibial tunnel is located on the lateral side of the tibial insertion of the PCL, which can reduce the impact between the graft and the medial side of the intercondylar fossa after reconstruction. 4. The popliteal artery mainly runs laterally, and the lateral tunnel is close to the posterior septum. The reflection of the posterior capsule is oriented medially, ensuring the safety of the operation.

PCL, posterior cruciate ligament.

**Table 2:** Advantages of Preserving the Remnant PCL

1. The mechanoreceptors existing in the remnant PCL can help with the recovery of the patient’s proprioception. 2. The remnant PCL can eliminate the closed-off effect. 3. Placing the graft above the remnant PCL can slow down wear of the graft and reduce the effect of the “killer turn.”

PCL, posterior cruciate ligament.

**Table 3:** Advantages of Ultrasound-Assisted Positioning

1. The ultrasound probe helps to locate the distal reflection of the posterior capsule and the posterior slope of the tibial plateau. 2. It can detect distance from the tibial nerve, popliteal artery, and popliteal vein, avoiding nerve and vascular injuries. 3. Ultrasound detection enables dynamic monitoring during surgery, which is safer and more convenient than intraoperative x-ray.

**Table 4:** Limitations of the Technique

1. When positioning the femoral tunnel, the surrounding tissues should be carefully cleared close to the edge of the cartilage to avoid damaging the PCL. 2. Due to the preservation of the remnant PCL, the problem of overfilling the graft may occur. Try to use a graft with a diameter of 8 mm as much as possible to avoid this problem. 3. During the tightening process, the knee should be in the “anterior drawer” position to ensure the graft’s tension, which will allow the patient to adapt to daily activities after surgery. 4. It is necessary to learn to use ultrasound technology to measure and position the tibial tunnel.

PCL, posterior cruciate ligament.

## Disclosures

J.D. reports financial support was provided by the 10.13039/501100001809National Natural Science Foundation of China and the 10.13039/501100008238Hebei Provincial Department of Science and Technology. All other authors (X.M., S.W., Z.L., Y.Z., Y.N.) declare that they have no known competing financial interests or personal relationships that could have appeared to influence the work reported in this paper.

## Supplementary Data

Video 1 After preoperative preparation and graft preparation, the anteromedial portal and anterolateral portal are created. The center of the medial opening of the femoral tunnel is 7 to 8 mm away from the distal side of the cartilage, and then the femoral tunnel is established. The PDS suture (Ethicon) is retained near the medial opening of the femoral tunnel. Then the double posteromedial portals are created. The low posteromedial portal is used as an observing portal and the high posteromedial portal is used as a working portal. The tibial tunnel is established with reference to the posterior capsule reflection and posterior septum. The femoral end of the graft is fixed by the adjustable looped titanium button, and the tibial end of the graft is fixed by the interference screw.
